# The motivational system of task values and anticipated emotions in daily academic behavior

**DOI:** 10.1007/s11031-021-09898-y

**Published:** 2021-07-03

**Authors:** Osman Umarji, Peter McPartlan, Julia Moeller, Qiujie Li, Justin Shaffer, Jacquelynne Eccles

**Affiliations:** 1 Yaqeen Institute for Islamic Research, Dallas, USA; 2 University of California, Irvine, Irvine, USA; 3 San Diego State University, San Diego, USA; 4 University of Leipzig, Leipzig, Germany; 5 New York University, New York, USA; 6 Colorado School of Mines, Golden, USA; 7 Australian Catholic University, Sydney, Australia

**Keywords:** Expectancy-value, Academic emotions, Intra-individual, Achievement motivation

## Abstract

This study integrates theories of achievement motivation and emotion to investigate daily academic behavior in an undergraduate online course. Using cluster analysis and hierarchical logistic regression, we analyze profiles of task values and anticipated emotions to understand expectations and completion of academic tasks over the duration of a week. Students’ task specific interest, opportunity cost, and anticipated satisfaction and regret varied across tasks and were predictive of both their expectations of task completion and actual task completion reported the following day. The results shed light on the important role of achievement motivation as situated and dynamic, highlighting the interplay between task priorities, task values, and anticipated emotions in academic task engagement.

## Introduction

Relations between university students’ domain-specific motivation and their long-term academic choices and behaviors have been extensively studied. However, success in a particular course depends on students completing individual tasks and assignments on a daily and weekly basis, and little is known about how students’ study intentions and motivation operate on a task-specific level. As Ajzen (1993) pointed out, the principle of aggregation (i.e., the sum of a set of multiple measurements is a more stable and representative estimator than any single measurement) does not explain behavioral variability across situations, nor does it permit prediction of a specific behavior in a given situation. Therefore, relying on domain-specific models of motivation (e.g., math motivation) may not reflect task-specific motivation (e.g., motivation for a particular math assignment) and behavior. As students approach academic tasks with varying levels of motivation, emotions, and priorities (Eccles et al., 1983; Eccles & Wigfield, 2020; Pekrun, 2006), understanding the interplay of these constructs on a task-specific level is fundamentally important in developing motivational theories of achievement (Dietrich et al., 2017). Furthermore, as technology has created new learning environments, motivational theories can be vehicles for understanding the affordances and challenges of these contexts.

The proliferation of online courses in universities, especially due to the COVID-19 pandemic, has provided a new context for learning, providing students with extensive control over the time and place that they complete course-related tasks. For students, this new context may be advantageous or problematic; favored or feared, depending on their motivation and ability to self-regulate their learning (Kizilcec et al., 2017; McPartlan et al., 2021; Means & Neisler, 2021). Prior research shows that ability beliefs (de Fátima Goulão & Menedez, 2015; Fryer & Bovee, 2016) and subjective task values (Chiu & Wang, 2008) are significant predictors of students' intentions to persist in e-learning. In this study, we integrate theories of motivation, emotions, goal-setting, and planned behavior to investigate daily academic intentions in an undergraduate online course and the extent to which motivation at the task level relates to daily task attainment.

When investigating intentions to complete course-related tasks at a daily level, the importance of goal hierarchies becomes apparent. With limited time in a day, students may *plan* an activity, but may fail to *complete* it if a confluence of self-regulation, achievement emotions, and task-values leads them to prioritize it lower than other activities. Additionally, students may disengage from a daily academic task for adaptive reasons (i.e., more urgent and important tasks that have come up) and reengage with the task at a more appropriate time. Literature on goal intentions (Gollwitzer, 1993), achievement emotions (Pekrun, 2006), the theory of planned behavior (Ajzen, 1991), and expectancy-value theory (Eccles et al., 1983) all provide insights into the process of task attainment, and we seek to synthesize these frameworks to better understand student behavior. As multiple theoretical perspectives use different terminology, we consider the terms goals and tasks as fundamentally referring to the same thing.

## Synthesizing theoretical frameworks

Numerous psychological models seek to explain motivated behavior at various levels. The underlying assumption of many of these models is that motivated behavior stems from the formation of an intention to engage in a particular task. These psychological models of motivation have focused on different facets of the motivational system, including the role of ability beliefs and subjective task values on domain-specific achievement choices and performance (Eccles et al., 1983), the relations between attitudes and intentions on actual behavior at the task-specific level (Ajzen, 1991), the role of emotions in achievement contexts (Pekrun, 2006), and the influence of self-regulation on goal pursuit (Gollwitzer et al., 2004). We seek to synthesize the focal constructs in each theory in order to better explain daily behavior in an achievement context at the person-level. We proceed to explain each theory of motivated behavior and the utility of weaving the focal constructs into an organized model to study daily academic behavior.

## Expectancy-value theory

According to Eccles’ expectancy-value theory (EVT), achievement-related intentions and behaviors are directly influenced psychologically by expectations of succeeding in a task (i.e., ability beliefs, such as self-efficacy and self-concept of ability) and the subjective task value associated with the task compared to other tasks. Subjective task value refers to the subjective aspects of a task that contribute to the increasing or decreasing probability than an individual will select and accomplish it (Eccles, 2005). Subjective task value is an emergent property of the task and determined by the perceived fit between the characteristics of the task itself and the actor (Eccles & Wigfield, 1995). Subjective aspects of a task emerge from (1) attainment value (the personal importance an activity has in fulfilling one’s identity or self-image); (2) intrinsic value^1^ (interest and enjoyment in task engagement); (3) utility value (usefulness of the task for other goals); and (4) the cost of engaging in the activity, which can be psychological, financial, or time and energy related.^2^

Most prior research on Eccles’ EVT has measured ability beliefs and subjective task values at the domain-specific level (e.g., math motivation) as predictors of future motivation (Umarji et al., 2021), academic intentions and aspirations (Gao & Eccles, 2020), college major selection (Umarji et al., 2018), college major persistence (Andersen & Ward, 2014), and achievement (Wigfield & Eccles, 2000). For example, typical survey items will ask students how good they think they are at math (self-concept) and how interested they are in the subject (interest value). However, Eccles and Wigfield have recently (2020) renamed their theory the situated expectancy-value theory, emphasizing the importance of contextual, situation-specific dynamics in ability beliefs and values, and some research has examined the fluctuating nature of ability beliefs and values (Dietrich et al., 2017, 2019; Parrisius et al., 2020; Tanaka & Murayama, 2014). Highlighting the situated, task-specific nature of ability beliefs and task-values is important, because different academic tasks, such as homework assignments and quizzes, within a course may have differing ability beliefs and value. Thus, on both a task-specific and domain-specific level, intentions, persistence, and achievement are distinct outcomes that are likely manifested through separate processes with unique predictors. Furthermore, intentions do not always translate into behavior. Within expectancy-value theory, the relative task value across available tasks is considered critical in linking intentions and behaviors, as intended tasks may be coopted by more valued tasks (Eccles & Wigfield, 2020). This intention-to-behavior-gap has received significant attention (Sheeran, 2002), and the theory of planned behavior (Ajzen, 2005) has addressed some of the mechanisms relating intentions to behavior.

## Theory of planned behavior & goals

When investigating motivation for daily academic tasks, motivational beliefs should be measured at the level of the specific task under consideration. Thus, when a specific behavior is to be predicted, such as task completion, the compatibility principle should be relied upon. The principle states that attitudes will better predict behavior if the specificity of a measured attitude matches the specificity of the behavior under consideration (Ajzen & Fishbein, 2005). Lack of utilizing this principle may be considered a limitation of some expectancy-value research that has measured attitudes at a domain level to predict various task-specific behaviors. Furthermore, intentions and actualization of the intentions must be disentangled in conceptualizing how motivation relates to daily academic tasks.

Task-related intentions are considered fundamental antecedents of task attainment. The formation of an intention is seen as being dependent on both the person’s attitude toward the behavior and the experienced normative pressures to executive it (Fishbein & Ajzen, 1975). When behavioral attitudes are positive and subjective norms favor the execution of a critical behavior, chances are high that the respective behavioral intention is formed. Students have many different desires and needs of what academic and non-academic behaviors to engage in daily, and some of these desires and needs may be in conflict with each other due to time constraints or the energy and effort required to realize them (Eccles, 2005). For example, on a particular day, a student may plan to go work, go to the gym, attend multiple class, and complete a number of required and optional school assignments. However, after attending multiple classes and going to work, the student may not have the time or energy to go to the gym or complete their school assignments. Additionally, daily tasks vary in priority and understanding the hierarchy of goal intentions is important in the study of goal attainment (Cropanzano et al., 1993; Eccles, 2005). Goal hierarchies refer to a mental system in which a person ranks a goal one above the other according to its perceived importance. Goal attainment has been found to be more likely to occur when based on personal value (e.g., autonomous) rather than controlled motives, such as feeling compelled due to internal or external pressures (Koestner et al., 2008). However, in an academic course, assignments are typically not negotiable and must be completed by a certain time and in a certain manner. Thus, goal setting in an academic context may operate differently than setting personal goals such as weight loss. Another important distinction in the study of goals can be made between goal intentions and goal expectations. Intentions refer to what a person intends to do, whereas expectations refer to how likely a person expects to do something (Warshaw & Davis, 1985). Expectations are theorized to capture unobserved factors that may cause a person to be unsuccessful in fulfilling their intention (Sheppard et al., 1988). Although expectancy-value research has investigated the predictors of academic goal intentions and aspirations, it has rarely considered the predictors of goal expectations. We believe incorporating goal expectations from the theory of planned behavior into EVT and educational psychological research is a valuable contribution in investigating the intention to behavior gap in educational settings.

## Linking emotions to goal-directed behavior

A link between the expectancy-value research, the research on academic emotions, and research on goals (e.g., Oettingen, 1996; Pekrun, 2006; Dirk, Schmidt, & Schmiedek, 2020) is the idea that individuals create expectations or fantasies about future events and their likelihoods of coming true, evaluate and predict how they would feel if that goal was successfully achieved or not, and then make decisions about the goal pursuit. In this thought, the fantasies are imaginations of the emotions that a person anticipates feeling in case of achieving -or not achieving- a future goal. The anticipation of emotions is considered key to the emotional goal system.

The research on academic emotions and the research on goal setting and goal pursuit agree in distinguishing between the evaluations and cognitions before, during, and after the pursuit of an academic goal and emphasize how different emotions and self-regulation strategies can be in these different phases (Heckhausen & Gollwitzer, 1987; Pekrun, 2006). Anticipated emotions of a possible future goal achievement are among the expected outcomes that are evaluated and weighted in the re-decisional phase, according to the rubicon model of action phases (Heckhausen & Gollwitzer, 1987; Pekrun, 2006). Anticipatory emotions reflect how positively one would feel if the goal were achieved, or how negatively one would feel if it were not (Bagozzi & Pieters, 1998). The accomplishment of a goal is expected to be followed by satisfaction, whereas the failure to accomplish a goal is expected to be followed by regret (Locke, 1996). Thus, satisfaction and regret appear to be retrospective emotions in the distinction between prospective, concurrent, and retrospective emotions (Pekrun, 2006). However, students can anticipate feeling satisfied in the case of a future goal accomplishment or they can anticipate feeling regret if they fail to achieve a goal due to a lack of proper preparation. Individuals who anticipate experiencing discontent or regret when they fall short of their goals will likely intensify their efforts in the goal pursuit (Gollwitzer, 1993), as regret is aversive, and individuals are motivated to avoid it (Zeelenberg et al., 1996). Anticipated regret refers to the extent of regret, tension, or distress a person would feel if they did not perform a particular behavior. It has strong associations with intentions to perform behaviors after other predictors have been controlled for (Richard et al., 1995). Anticipating regret about failing to perform a behavior might bind people to their intentions, such that participants who both intend to perform a behavior and anticipate considerable regret if they do not perform it, should exhibit greater intention behavior consistency than participants with equivalent intentions who do not anticipate regret (Sheeran & Orbell, 1999).

Emotions overlap with some aspects of task value (e.g., intrinsic value overlapping with the emotions of joy and interest, and emotional costs overlapping with negative emotions). For example, anticipated regret may also be considered an emotional cost, which is a negative component of task values (Perez et al., 2014). The subjective value of a task may also determine the strength of anticipatory emotions, as well as of goal pursuit effort, since emotions and goal pursuit effort are expected to rise with the subjective importance (i.e., task value) of a task. The accomplishment of a goal with a high subjective value is expected to be followed by satisfaction, whereas the failure to accomplish a subjectively valuable goal is expected to be followed by regret (Locke, 1996). Goals serve as the reference standard when individuals evaluate whether to feel satisfied versus dissatisfied (Mento et al., 1992).

Task values and anticipatory emotions can be crucial drivers of the motivation to choose a certain goal and to exert effort in the goal pursuit (e.g., Dietrich et al., 2017; Oettingen & Mayer, 2002). Many of the experiments by Oettingen and colleagues demonstrate that anticipated positive feelings upon a future goal achievement contribute to the motivational force helping people to attain that goal, but only if complemented by additional cognitive planning steps, such as identifying and avoiding obstacles and making specific action plans (Adriaanse et al., 2010; Oettingen, 2015; Oettingen & Mayer, 2002; Oettingen et al., 2005). The effect of emotions on performance likely depends on the mechanisms facilitated by the emotion and their interactions with task demands. Positive emotions may focus attention, foster interest, and promote self-regulation of a task (Pekrun & Stephens, 2009).

## Self-regulation

Goal intentions, task-values, and anticipated emotions do not automatically lead to goal attainment. The actions required to accomplish one’s goal must ensue after the intention has been made. Based on the model of Pintrich and De Groot (1990), self-regulation behaviors consist of three components: students’ use of cognitive strategies, their metacognition, and their management of academic resources (i.e., time and learning environment management, effort regulation, and help-seeking). Whereas motivation, goal setting, and emotion may all be important for initially developing goals, self-regulation behaviors then become important determinants of whether that goal will be attained by regulating students’ cognition, motivation, effort, and behavior as they actually pursue those goals (Pintrich, 2000). For example, effort regulation has been found to be negatively associated with procrastination (Ziegler & Opdenakker, 2018), and continuous strategic planning has proven to be especially predictive of attainment in online courses (Kizilcec et al., 2017). Yet, even once goals are set, students’ use of cognitive strategies to regulate pursuit of those goals can certainly still be linked to motivation and emotion. The act of imagining positive or negative emotional outcomes may initiate self-regulated behavior (Boekaerts, 2011), and a person’s self-regulation behavior (e.g., metacognition) can reinforce their motivational belief that they can successfully complete the task throughout the process (Efklides, 2011), emphasizing the importance of understanding how these processes often operate together when considering daily behavior.

## Applying theories of student behavior to online learning environments

As the prevalence of online learning has grown, these theories have also proven useful in understanding student behavior in online contexts. Recent research using these theories has shown that studying motivation (Edwards, 2020; Rosenzweig et al., 2019), emotions (Artino & Jones, 2012; Lehman et al., 2012), planned behavior (Chu & Chen, 2016; Ndubisi, 2006), and self-regulation (Kizilcec et al., 2017; Yeh et al., 2019) can produce meaningful explanations for online students’ behavior just as it can in the face-to-face environments in which these theories were developed (Noteborn et al., 2012). Some research has even explored relationships between these topics specifically among online learners, including emotion, ability beliefs, and task value (Marchand & Gutierrez, 2012; Noteborn et al., 2012). In addition, online learning studies investigating self-regulation have explored its individual relationships with emotion (Artino & Jones, 2012), task value (Sansone et al., 2012), and planned behavior (Lung-Guang, 2019).

Theories of motivation and self-regulation, especially, have become important tools for understanding student achievement in online contexts. Expectancy-value theory has been useful in identifying that for certain students, selecting into online courses may be a signal of motivational differences. Selecting online versions of courses is often done due to either lower interest in the course relative to other courses (Jaggars, 2014; McPartlan et al., 2021), or recognizing the high costs of balancing the course with other responsibilities (McPartlan et al., 2021; Vanslambrouck et al., 2018). Meanwhile, studies on self-regulation have long noted the fact that online students have significantly more autonomy of when and how to engage with course content, making goal setting, time management, effort regulation, metacognition, and critical thinking crucial predictors of their academic performance (Broadbent & Poon, 2015; Chang et al., 2013; Handoko et al., 2019; Lynch & Dembo, 2004). As this research demonstrates, online learning environments may initially be chosen by students who are already facing motivational barriers, and thereafter may not be structured to support students’ self-regulation as well as well as face-to-face courses. Overall, theories of motivation, emotion, and self-regulation developed to study student behavior in traditional contexts have seemed to show a fundamental compatibility with online contexts (e.g., Daniels & Stupinsky, 2012). In online environments, however, understanding how students’ motivation and self-regulation is impacting their behavior may be especially consequential for supporting students’ achievement.

## Person-centered research on motivated behavior

Based upon the prior discussed theories and constructs related to motivated behavior, situational heterogeneity of motivation is likely present both within and across students. For example, anticipated emotions, interest, and opportunity cost may differ for numerous reasons depending on the type of activity (e.g., reading or completing a homework assignment), the valued alternatives available to an individual that day or moment (e.g., opportunities to hang out with friends or the need to study for another exam), or a host of other situation or person specific reasons. Person-centered approaches that investigate such heterogeneity within or between people and moments have gained popularity due to considerations of increased ecological validity by not assuming ergodicity in psychological processes and due to the finer-grained details that they allow (Howard & Hoffman, 2018). The assumption of ergodicity assumes that the structures of interindividual and intraindividual variation are asymptotically equivalent, and violations of this principle may lead to incorrect inferences, including the ecological fallacy. The ecological fallacy occurs when statistical inferences from groups are inappropriately generalized to individuals (Fisher et al., 2018). To avoid these concerns, recent research in the field of educational psychology has utilized person-centered approaches to understand heterogeneity in undergraduate science courses and students’ patterns of engagement across contexts (Fong et al., 2021; Robinson et al., 2017), situational fluctuations in ability beliefs and values (Dietrich et al., , 2017, 2019), and academic emotions in adolescence (Ganotice et al., 2016; Moeller et al., 2018). These studies consistently identified unique profiles with respect to key motivational constructs. In a study focusing on profiles of ability beliefs, values, and costs in an undergraduate science course, the authors found four profiles that included low motivation situations, highly motivating situations, low cost motivation settings, and motivating but costly situations (Dietrich et al., 2019). Although the authors investigated associations between global motivational dispositions, they did not investigate how these profiles associated with subsequent behavior on a task. Another study by Robinson and colleagues (2017) found four affective profiles of situations in a college anatomy course (positive activated, positive deactivated, negative activated, and negative deactivated) and found that behavioral and cognitive engagement in these situations mediated the effects of the profiles on a course exam. These studies provide support for investigating the heterogeneity in experiences depending on the situation or task, yet it is important to note that each of these studies considered profiles within a set of constructs from a single theory (e.g., expectancy-value and control-value) only.

## Current study

**Fig. 1:**
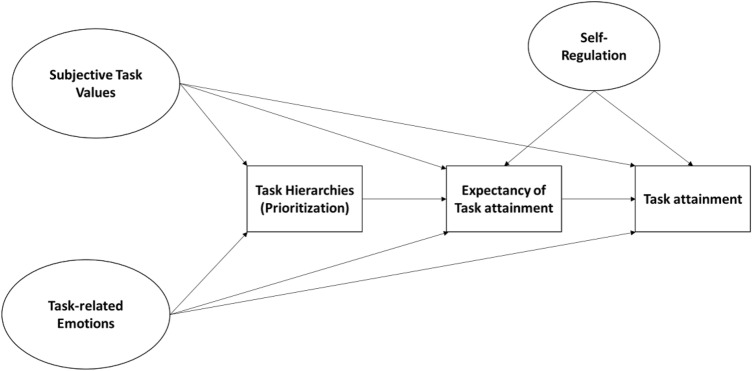
Conceptual model of daily task motivation

In the present study, we build upon the task motivation literature by integrating expectancy-value, control-value, goal setting, and the theory of planned behavior perspectives. We propose a model describing the motivations and emotions involved in daily learning processes, building on recent advancements in expectancy-value theory (Eccles & Wigfield, 2020; Moeller et al., 2020). We begin with the idea that task values are situated and fluctuate between tasks. We then integrate control-value theory that posits anticipated emotions influence task engagement. We also include the role of task prioritization from goal setting theories and goal expectations from the theory of planned behavior. Our study contributes to the literature in a number of ways**.** First, we study task motivation in the novel context of an asynchronous online course, which allows students the autonomy to plan and engage with the course material on their own time. This allows us to investigate motivation in the learning settings in which it naturally occurs. Second, we investigate the co-occurrence of subjective task values (interest and opportunity cost) and anticipated emotions intra-individually and across multiple tasks to understand heterogeneity in these motivational profiles. Third, we investigate the associations between motivational profiles and both task expectations and task attainment, as a first step to linking theories about achievement-related motivation and emotions with theories about goal-related aspects of self-regulation. See Fig. 1 for a conceptual model of the hypothesized relations between constructs. We seek to answer the following research questions:

1. What motivational profiles of subjective anticipated task values (e.g., interest and cost) and anticipated emotions (e.g., satisfaction and regret) cooccur within students across tasks?
2. To what extent do these motivational profiles relate to expected and actual task completion?
3. To what extent do these motivational profiles, effort regulation and task hierarchy relate to expected and actual task completion?

## Method

### Participants

The participants in this study were recruited from an undergraduate, summer, online class at a large public university in the Southwest United States, in which students are roughly 40% Asian, 26% Hispanic, 20% White, and 4% Black. Also, roughly 60% are women, 50% are from first-generation backgrounds, and 40% are from low-income backgrounds. Of the 147 students in the course, this study utilizes a subsample of students (n = 101) who completed daily diary surveys during the third week of the course.

### Setting

The class was an online, elective course on the biology and chemistry of food and cooking, designed for non-biology majors. The course was offered in an accelerated five-week period (as compared to the typical 10-week quarter), and was considered a “high structure” course, in which students were assigned deadlines for completing several different assignments each week (Eddy & Hogan, 2014; Lieu et al., 2017). The material was delivered through a combination of lecture videos (12 per week of roughly eight minutes each) and a class textbook (three chapters per week). Students’ overall grade comprised several graded assignments. The three exams (75% of overall grade) were multiple choice, each covering a third of the course material. Pre-lesson quizzes (9%) were completed by students after reading their textbooks and completing optional reading guides (word documents with questions to help students navigate their textbooks) (Lieu et al., 2017). These were intended to help students assess their knowledge prior to watching class videos. Weekly review quizzes (8%) were completed by students to help them assess their understanding from the prior week and thus to help them manage their studying in advance of each exam. Discussion board posts (6%) were completed by students after watching pre-recorded lesson videos. Students were asked to expand upon what they just learned in by connecting the content to their daily lives, asking follow-up questions, or analyzing data from the literature. Students were also given 2% of their grade for completing course evaluations, and two surveys at the beginning and end of the course, respectively.

### Procedure

The instructor informed students they would receive course credit for completing either of two surveys related to course motivation in the first and sixth weeks of the course (pre and post). The instructor then informed students that a research team would be offering a financial incentive for completing up to ten additional surveys throughout the term. Students were offered $3 for each completed survey, as well a $5 bonus for students who completed at least eight of the 10 surveys. Of these, four surveys were administered at the start of every week (weeks two to five), and six surveys were administered each morning during week three. Surveys were administered in Qualtrics. In addition to email notifications, students who opted to provide their phone numbers in the pre-survey were sent text messages with links to personalized online surveys for the daily and weekly surveys (This study was IRB approved: HS# 2015-2225).

### Measures

#### Daily activity

Students were asked “Please list all the [course-related/other] activities you plan to accomplish today,” with up to five open-entry spaces for course-related activities and five additional open-entry spaces for “other” activities, which were explained as “(e.g., working for pay, caring for family members, taking another course, playing sports, completing home projects, etc.”). Responses were coded for type of activity (e.g., reading, watching videos, take a quiz). Two research assistants independently coded each activity, achieving strong interrater reliability (*k* = 0.83).

#### Task importance rank

Students were asked to rank order all their planned daily activities from the most important to least important, with one being the most important activity of the day.

#### Interest

Interest for each daily course activity was assessed with the single item “how interesting is this course-related activity” on a Likert scale from 1 = not at all interesting to 7 = very interesting.

#### Opportunity cost

One item was used to assess opportunity cost for each daily course activity (item, “how much will you have to give up to complete this activity?” on a Likert scale from 1 = nothing to 7 = a lot).

#### Anticipated regret

One item was used to assess anticipated regret for each daily course activity (item, “How much regret will you feel if you do not complete this activity?” on Likert scale from 1 = none at all to 7 = extreme).

#### Anticipated satisfaction

One item was used to assess anticipated satisfaction for each daily course activity (item, “How much satisfaction will you feel if you complete this activity?” on Likert scale from 1 = none at all to 7 = extreme).

#### Expectation of activity completion

One item was used to assess expectancy of activity completion (item, “to what extent do you expect to complete this activity?” on a Likert scale from 1 = not at all to 7 = completely). Due to the skewed distribution of the response, where the majority of activities were rated a 7, the item was recoded into a dichotomous variable of whether or not the student absolutely expected to complete the activity or not. Absolute expected complete was coded as 1 if the response was 7. Otherwise, it was coded as 0.

#### Actual activity completion

One item was used to assess actual activity completion on the following day (item, “to what extent did you complete this activity?” on a Likert scale from 1 = not at all to 7 = completely). Due to the skewed distribution of the response, where the majority of activities were rated a 7, the item was recoded into a dichotomous variable of whether or not the student absolutely completed the activity or not. Absolute completion was coded as 1 if the response was 7. Otherwise, it was coded as 0.

#### Effort regulation

Effort regulation (ER) was measured prior to the start of the course in the pre-survey. Items from the Motivated Strategies for Learning Questionnaire (Pintrich et al., 1993) were used to assess students’ self-reported ability to regulate their effort (Cronbach’s α = 0.79) related to academics (sample item, “Even when course materials are dull and uninteresting, I manage to keep working until I finish”, measured on a Likert scale from 1 = strongly disagree to 5 = strongly agree).

### Attrition and missing data

The data in this study include a complex pattern of complete and missing data. Students were able to complete up to five course-related and five non-course related activities daily. If students did not respond to the daily survey, then the data were treated as missing. However, if the student put in at least one activity, the data was considered complete. Students who participated in the daily surveys had higher final course grades than those who did not participate in the daily surveys. However, t-tests showed that there were no significant differences between daily survey participants and non-participants in any other key motivational constructs, including course importance, course interest, effort regulation, and self-efficacy. Of the 101 students who participated in the daily surveys, 26 students were dropped from our analysis sample due to missing data at the person level, as HLM drops cases with missing values at level two (Raudenbush & Bryk, 2002). The final analysis sample included 75 students with valid daily survey data (i.e., they responded to at least one daily survey) and baseline data on effort regulation, yielding 561 total daily tasks reported. The average number of task-specific observations per student was 7.5. Based on HLM power literature, 80 level-2 units have been suggested to reach the threshold power of 0.80 for level-1 fixed effects with no slope variance and small level-1 sample sizes (Schoeneberger, 2016).

### Analysis plan for research questions 1 & 2

Cluster analysis was used to investigate patterns of task values (e.g., interest and cost) and anticipated emotions (satisfaction and regret). Cluster analysis allows for classifying each task into homogeneous subgroups with respect to the patterns of task values and emotions reported by the student by maximizing within-cluster homogeneity and between-cluster heterogeneity (Magnusson & Törestad, 1993; Wormington et al., 2012). Raw scores for interest, cost, regret, and satisfaction were used for each task, as standardizing introduces numerous problems with interpreting the data for longitudinal studies, especially in profile analyses, as the z-score represent rank in relation other students, not the extent to which an item was endorsed by a student (Moeller, 2015). A multi-step analysis was carried out using ROPSTAT (Vargha et al., 2015), a statistical package for person-centered analyses. The following steps were performed:

1. Preparatory steps of removing outliers;
2. Hierarchical cluster analysis followed by K-means relocation clustering.
3. Random sample validation procedure to confirm cluster stability and reliability.

Multivariate outliers were identified using the RESIDAN method (Bergman, 1988b), which identifies outliers prior to clustering. Hierarchical clustering methods are sensitive to outliers that may bias the hierarchical structure at any level of merging, and the cutoff point was a squared Euclidean distance greater than 0.7 (Bergman et al., 2003). Three outliers were removed from the analysis sample.

After the preparatory steps were completed, cluster analysis was performed using Ward’s method, a hierarchical agglomerative method that initially assigns each case to its own cluster and step-by-step the most similar clusters are joined together, eventually resulting in one cluster with all cases (Clatworthy et al., 2005). Ward’s method is based on squared Euclidian distances to create a similarity/dissimilarity matrix, aiming to minimize the within-cluster sum of squares (Wormington et al., 2012). Additionally, it makes no assumptions about the distribution of the data being used. In order to determine the most suitable cluster solution, both a priori theorizing of clusters and statistical considerations based on the percent of variance explained were considered. The error sum of squares (ESS), a measure of cluster heterogeneity, and the explained error sum of squares (EESS) were calculated for all possible cluster solutions.

EEES = 100*((TotalESS-ESS_of_the_given_cluster_solution)/TotalESS).

An EESS value of 100 implies perfect cluster homogeneity, whereas 0 implies the complete absence of cluster homogeneity (Bergman et al., 2003). ESS values were plotted against EESS values to display an array of possible cluster solutions based on how much additional error was included by reducing a cluster from the previous solution. This analysis was carried out at every wave independently, as it is possible that a different number of clusters would emerge at different developmental stages.

K-means clustering was performed to fine-tune cluster homogeneity by reassigning cases to the optimal cluster. In K-means clustering, the number of clusters is chosen before relocation using the initial hierarchical method. Centroids (i.e., profiles of means for the variables in the clusters) from the Ward’s analysis were used as starting points, and all cases within a certain distance of the centroid became assigned to that cluster until all cases were assigned (Wormington et al., 2012). The K-means analysis reduced the homogeneity coefficient of the clusters at each wave, confirming that case relocation was appropriate. Cluster stability and reliability was tested by drawing a random split of the sample and confirming that similar clusters appeared. After all cluster solutions were completed, cross-tabulations with adjusted standardized residuals were used to test for differences in cluster membership and expected and actual task completion.

### Analysis plan for research question 3

Hierarchical logistic regression was used to predict the likelihood of expected and actual task completion based on cluster membership, effort-regulation, and task importance rank. Two models were estimated, one for expected task completion and one for actual task completion. Repeated measures of cluster membership and goal hierarchy (level-1) were nested within students (level-2). Effort regulation (level-2) was grand-mean centered, allowing us to analyze inter-individual differences (Enders & Tofighi, 2007).

To address missing data issues, restricted maximum likelihood (REML) was used for estimation of variance and covariance components. REML estimates of variance components account for the uncertainty of the fixed effects. Full maximum likelihood estimates were computed as a robustness check, and the results were very similar. All analyses were estimated using HLM 7 software using robust standard errors.

The final model for each outcome was:

#### Level-1 Model

$$\begin{gathered} {\text{Prob}}({\text{Task}}\;{\text{Completion}}_{{ti}} = {\text{1}}|\pi _{i} ){\text{ }} = \phi _{{ti}} ~ \hfill \\ \;\;{\text{log}}[\phi _{{ti}} /({\text{1 }} - \phi _{{ti}} )]{\text{ }} = {\text{ }}\eta _{{ti}} \hfill \\ \;\;\eta _{{ti}} = \pi _{{0i}} + \pi _{{1i}} *(C1\_C_{{ti}} ){\text{ }} + \pi _{{2i}} *(C3\_C_{{ti}} ){\text{ }} + \pi _{{3i}} *(C4\_C_{{ti}} ){\text{ }} + \pi _{{4i}} *(C5\_C_{{ti}} ){\text{ }} + \pi _{{5i}} *(C6\_C_{{ti}} ) \hfill \\ + \pi _{{6i}} *(task\;importance\;rank_{{ti}} ) \hfill \\ \end{gathered}$$

#### Level-2 Model

$$\begin{gathered} \pi _{{0i}} = \beta _{{00}} + \beta _{{01}} *(ER_{i} ){\text{ }} + r_{{0i}} \hfill \\ \pi _{{1i}} = \beta _{{10}} ~~ \hfill \\ \pi _{{2i}} = \beta _{{20}} \hfill \\ \pi _{{3i}} = \beta _{{30}} ~ \hfill \\ ~\pi _{{4i}} = \beta _{{40}} \hfill \\ \pi _{{5i}} = \beta _{{50}} ~ \hfill \\ ~\pi _{{6i}} = \beta _{{60}} \hfill \\ \end{gathered}$$

where η_*ti*_ represents the outcome (expected/actual task completion) of the *i*th student on the *t*th task measured and *e*_*ti*_ represents the level-1 residual. The parameters, *β*_*10*_* to β*_*50*_, represent the estimates of the likelihood of expected and actual completion by each cluster, relative to the average cluster, C2. The parameter, *β*_*60*_, represents the association between task importance rank and the likelihood of expected/actual task completion. *β*_*01*_ represents the effect of effort regulation on expected/actual task completion. Heterogeneity in the intercept is captured by the random effects, *r*_*0i*_.

## Results

### Preliminary Findings

Before presenting our findings on the patterns of task values and anticipated emotions and their association with task attainment, we provide a summary of the descriptive findings as a necessary backdrop to the forthcoming analyses. Means and standard deviations are provided in Table 1. Students expected to complete 55% of all their daily tasks but reported completing approximately 43% of them. Of all the task-related motivational constructs, anticipated emotions of regret and satisfaction were the highest on average, whereas opportunity cost had the lowest mean but the largest standard deviation. Correlations for all study variables are found in Table 2. Task values and anticipated emotions were associated with expectations and actual task completion, with the exception that interest value was not associated with actual task attainment. From this correlational approach, the relatively weak relationships among study variables reaffirmed the fact that ratings of implementation intentions (expected completion), motivation, and emotion for different tasks can co-occur in a variety of different ways, reinforcing the appropriateness of a pattern-centered approach for our remaining analyses.

**Table 1:** Means, standard deviations, and ranges for all study variables

	*Mean*	*SD*	*Min*	*Max*
Expected completion (dichotomized)	0.55	0.50	0	1
Expected completion	6.20	1.21	1	7
Actual completion (dichotomized)	0.43	0.50	0	1
Actual completion	5.16	2.27	1	7
Interest	4.90	1.38	1	7
Cost	3.76	1.70	1	7
Anticipated regret	5.66	1.49	1	7
Anticipated satisfaction	5.78	1.50	1	7
Effort Regulation	3.48	0.64	2.43	5
*N*_tasks_	561

**Table 2:** Correlations of all study variables

	Expected	Actual	Interest	Cost	Regret	Satisfaction	Task rank	ER
Expected	-							
Actual	0.44*	-						
Interest	0.09*	0.03	-					
Cost	-0.14*	-0.15*	0.11*	-				
Regret	0.29*	0.23*	0.25*	0.09*	-			
Satisfaction	0.14*	0.13*	0.47*	0.14*	0.36*	-		
Task rank	0.22*	0.21*	0.08	-0.07	0.18*	0.10*	-	
ER	0.20*	0.14*	0.06	0.12*	0.11*	0.19*	-0.12*	-

All variables are repeated measures except for ER = Effort Regulation

* p < 0.05

### Clusters of task values and anticipated emotions

The initial results from Ward’s hierarchical method revealed that a cluster solution between six and nine clusters could be considered by analyzing the ESS and EESS plots. After investigating the scree-plot, variance explained, and the specific clusters in each solution, we determined that a six-cluster solution best fit and explained the data parsimoniously. Every cluster solution beyond the six-cluster solution began to break one distinct cluster into subgroups that were not theoretically meaningful. Larger solutions also did not explain substantially more variance. Additionally, K-means clustering was used to relocate cases, correcting preliminary classification and increasing cluster homogeneity. The final six-cluster solution accounted for 67.6% of the variance, above prior used thresholds of 50% or 67% (Hayenga & Corpus, 2010; Wormington et al., 2012).

**Fig. 2:**
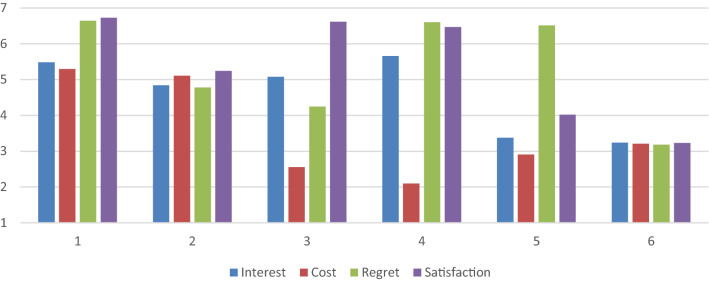
Six-cluster solution for subjective task values and emotions profiles

We describe the clusters in terms of the extent to which interest (as an emotion & aspect of task value), opportunity cost, anticipated regret, and anticipated satisfaction were high, medium, or low relative to other clusters. Cluster means and homogeneity coefficients are displayed in Table 3 and Fig. 2 illustrates clusters visually. Motivational clusters were labeled as Cluster 1: High emotions/high cost (*n* = 171; 30%), Cluster 2: medium emotions/medium cost (*n* = 87; 15%), Cluster 3: low cost/high satisfaction (*n* = 83; 15%), Cluster 4: high emotions/low cost (*n* = 124; 22%), Cluster 5: high regret (*n* = 47; 8%), and Cluster 6: low emotions/low cost (*n* = 54; 10%). The high emotions/ high cost cluster (1) and the low emotions and low cost cluster (6) refer to daily tasks that were considered high or low in all four constructs of interest, cost, anticipated regret, and anticipated satisfaction. The low cost/high satisfaction cluster (3) referred to daily tasks where opportunity cost was considered very low, interest was medium, anticipated regret was somewhat low, but anticipated satisfaction was very high. The high emotions/low cost cluster (4) referred to daily tasks that were quite high on the three emotions of interest, anticipated regret, and satisfaction, but were low on opportunity cost. The high regret cluster (5) was low on interest, cost, and anticipated satisfaction, but high on regret only.

**Table 3:** Cluster centroids, size, and homogeneity coefficients

Name	Interest	Cost	Regret	Satisfaction	Cluster Size	HC
1. High emotions/high cost	5.49	5.30	6.64	6.73	171	1.22
2. Medium emotions/medium cost	4.84	5.11	4.78	5.24	87	1.86
3. Low cost/high satisfaction	5.08	2.56	4.25	6.62	83	1.88
4. High emotions/low cost	5.66	2.10	6.61	6.47	124	1.26
5. High Regret	3.38	2.91	6.51	4.02	47	2.08
6. Low emotions/low cost	3.24	3.21	3.19	3.23	54	2.52

*HC* Homogeneity coefficient

### Cluster membership and task completion

Cross tabulation and adjusted standardized residual analyses (*ASR*) were conducted to determine if cluster membership alone was related to expected and actual task completion. Separate analyses were done for expected task completion and actual task completion reported the following day. The results of the chi-squared analyses are available in Table 4. Tasks in the high emotions/high cost cluster (1) were expected to be completed significantly more than expected by chance (*ASR* = 3.38, *p* < 0.001), as were tasks in the cluster of high emotions/low cost (*ASR* = 5.00, *p* < 0.001), whereas tasks that were low emotions/low cost (cluster 2; *ASR* = − 2.96,* p* < 0.001) and medium emotions/medium cost (cluster 2; *ASR* = − 0.6.33,* p* < 0.001) were significantly less likely to be completed than expected by chance (e.g., these clusters were underrepresented in expected task completion). Regarding actual task completion, the medium emotions/medium cost tasks (cluster 2; *ASR* = − 0.3.35,* p* < 0.001) and the low emotions/low cost tasks (cluster 6; *ASR* = − 4.12, *p* < 0.001) were completed significantly less than expected by chance, whereas the high emotions/low costs tasks (cluster 4; *ASR* = − 0.5.24,* p* < 0.001) were more likely to report completing a task than expected by chance. Low cost/high satisfaction (cluster 3) and high regret (cluster 5) completed tasks as often expected by chance (*p* > 0.05).

**Table 4:** Chi-squared test for Cluster Membership and Task Completion

Cluster	Expected	
	0	1
High emotions/high cost		
Observed	70	135
ASR	− 3.381	3.381
Medium emotions/medium cost		
Observed	85	36
ASR	6.331	− 6.331
Low cost/high satisfaction		
Observed	48	45
ASR	1.558	− 1.558
High emotions/low cost		
Observed	47	124
ASR	− 4.995	4.995
High regret		
Observed	36	48
ASR	− 0.246	0.246
Low emotions/low cost		
Observed	40	25
	2.963	− 2.963

Cluster	Actual	
	0	1
High emotions/high cost		
Observed	84	74
ASR	− 0.317	0.317
Medium emotions/medium costs		
Observed	75	35
ASR	3.247	− 3.247
Low cost/high satisfaction		
Observed	45	30
ASR	1.07	− 1.07
High emotions/low cost		
Observed	47	89
ASR	− 5.238	5.238
High regret		
Observed	34	38
ASR	− 1.275	1.275
Low emotions/low cost		
Observed	41	9
ASR	4.115	− 4.115

Overall *χ*^2^ = 71.14, df = 5, *p* < 0.000

Overall *χ*^2^ = 47.87, df = 5, *p <* 0.000

*ASR* Adjusted standardized residuals

### Hierarchical logistic regression

To better understand differences in task completion based on task-specific motivational clusters, in addition to task importance rank (e.g., relative hierarchy of task importance) and effort regulation, we conducted a hierarchical logistic regression for expected and actual task completion. The results are detailed in Table 5. Each cluster was dummy coded, with the reference group for the analysis being the medium emotions/medium cost tasks (cluster 2). This cluster was chosen as the reference group as it was considered the most average of all the clusters with respect to the four variables. We begin by explaining the results for expected task completion. At the task-level, task importance rank was positively associated with expected task completion (*OR* = 1.37, *p* < 0.001), meaning that the higher a task was ranked within the hierarchy of all daily tasks for a student, the more likely a student expected to complete that task. Relative to the reference cluster (medium emotions/medium cost), a task in Cluster 1(high emotions/high cost) or Cluster 4 (high emotions/low cost) was significantly more likely to be rated by a student as a task that they expected to fully complete (Cluster 1: *OR* = 3.97, *p* < 0.001; Cluster 4: *OR* = 5.52, *p* < 0.001). Cluster 3 (low cost/high satisfaction), Cluster 5 (high regret), and Cluster 6 (low emotions/low cost) did not significantly differ from the reference cluster (medium emotions/medium cost) in the likelihood of expected task completion (*p* > 0.05). At the person level, self-reported effort regulation was positively associated with expecting to complete a task (*OR* = 1.37, p < 0.05).

**Table 5:** Hierarchical logistic regression models of expected and actual task completion

	Expected task completion		Actual task completion	
Fixed effect	*Odds Ratio*	*SE*	*Odds Ratio*	*SE*
Intercept	1.20	(0.34)	1.25	(0.40)
Effort regulation	1.68*	(0.21)	1.66*	(0.23)
Cluster 1: high emotions/high cost	3.97*	(0.40)	1.71	(0.35)
Cluster 3: low cost/high satisfaction	1.99	(0.44)	1.40	(0.39)
Cluster 4: high emotions/low cost	5.52*	(0.39)	2.57*	(0.38)
Cluster 5: high regret	1.79	(0.43)	1.56	(0.45)
Cluster 6: low emotions/low cost	0.97	(0.54)	0.54	(0.39)
Task rank	1.37*	(0.10)	1.40*	(0.11)
	*Variance*	*χ2 df* = 68	*Variance*	*χ2 df* = 68
Random intercept	1.00*	151.83	1.00*	151.84

Results are presented as odds ratios. SE = Standard error, which are based on the log odds. *p < *.05*

For actual task completion, task importance rank was also positively associated with actual task completion (*OR* = 1.40, *p* < 0.001). Relative to the reference cluster, a task in the high emotion/high cost cluster was not more likely to be completed (*p* > 0.05), after controlling for effort regulation and task importance rank. However, a task in the high emotions/low cost cluster was more likely to be completed than a task in the medium cluster (*OR* = 2.57, *p* < 0.05), after controlling for effort and task importance rank. All other clusters did not significantly differ from the medium cluster. Self-reported effort regulation was positively associated with actually completing a task (*OR* = 1.66, p < 0.05).

### Post-hoc analyses

The high emotions/high cost cluster (1) and high emotions/low cost cluster (4) were both overrepresented in expectations of task completion. However, only tasks in the high emotions/low cost cluster (4) were completed more often than expected. As a robustness check, we performed a test of proportions to confirm whether the difference between the two clusters was statistically significant. There was no difference in the proportion of expected task completion (*z* = − 1.19, *p* = 0.23) between the two clusters, but there was a significant difference in the proportion of actual task completion (*z* = − 2.55, *p* = 0.01).

We also performed a crosstab analysis of cluster by academic task type. This was done to better understand the association between motivational clusters and specific types of academic tasks that students engaged with throughout the course. Coding suggested there were six types of academic tasks that students reported engaging with: (1) quizzes/ exams, (2) textbook reading, (3) textbook study guides, (4) watching lecture videos, (5) discussion posts, and (6) review. The overall chi-squared test was significant (*p* < 0.001) and results are available in Appendix A. The high emotions/high cost cluster (1) was underrepresented in reading study guides and discussion posts. The high emotions/low cost cluster (4) was overrepresented in discussion posts. The high regret cluster (5) was overrepresented in quizzes and exams, whereas it was underrepresented in watching lecture videos. The low emotions/low cost (6) was underrepresented in quizzes and exams, whereas it was overrepresented in discussion posts.

## Discussion

The current study integrated aspects of expectancy-value theory, control-value theory, the theory of planned behavior, and goal setting theories in order to better understand daily task motivation and task achievement for students in an undergraduate online course. We have contributed to the literature by synthesizing psychological theories of behavior using a person-centered approach that sheds light on intraindividual and interindividual differences in motivation and its relationship with task attainment on a daily level. Significant heterogeneity in cluster membership was observed, suggesting that the motivational system of values and emotions substantially varies between tasks within and between students. Moreover, clusters were predictive of both expected task completion and actual task completion, suggesting that the motivational system of task values and emotions relates to daily academic task behavior, in line with similar, recent findings from variable-centered approaches (e.g., Ketonen et al., 2018; Theobald, Breitwieser, Murayama, & Brod, 2021).

Predicting task expectations and completion by motivational and emotional profiles aligns well with expectancy-value theory. Prior EVT research typically investigates intentions and achievement, and typically ignores the intention to behavior gap. Our findings contribute to new insights in considering how motivational profiles related to task expectations and task attainment, with implications for considering students’ metamotivational control. Take the two largest clusters, for example. High emotion/high cost tasks (Cluster 1) were perceived as very interesting, requiring students to give up a lot in order to complete, and elicited dual anticipated feelings of satisfaction if completed and regret if not completely. High emotion/low cost tasks (Cluster 4) were perceived to be just as interesting and emotionally-loaded but were tasks that students did not believe they would have to give up much to complete. These tasks (Cluster 1 & 4), which comprised over 50% of all reported tasks, were the ones students expected they would be especially likely to complete; suggesting the combination of high interest, anticipated satisfaction, and anticipated regret they had in common indeed aggregate up to excite students towards action. However, only the high emotion/low cost tasks were especially likely to actually be completed, underscoring the role that cost plays in the intention to behavior gap despite the presence of other motivating perceptions. More specifically, it is possible that although students planning high emotion/high cost tasks were aware of the high opportunity cost, the strength of the other motivating factors made them overly optimistic about finding the time to do it. In other words, perhaps they underestimated the actual cost of a perceived costly task. Alternatively, perhaps students disproportionally weighed their high level of interest and anticipated emotions toward these tasks above their considerations of the time investment required. This dilemma may relate to motivation regulation and metamotivational control, which refers to the process by which a student attempts to maintain the level and type of motivation required to optimally pursue a goal (Miele & Scholer, 2018).

Our pattern-centered approach also helps contextualize previous research on the effects of anticipated regret by showing that high anticipated regret alone is not enough to make students especially likely to complete a task. Previous variable-centered and interindividual approaches have found that anticipated regret is associated with stronger intentions and behavior (Gollwitzer, 1993; Richard et al., 1995; Sheeran & Orbell, 1999). While this may be true when all else is held constant, our high regret cluster (Cluster 5) shows that students don’t expect they’ll be especially likely to complete tasks if high anticipated regret is not accompanied by substantial interest or anticipated satisfaction. Tasks fitting this high regret profile can be quite consequential, making it easy to understand why students perceived such high regret. In our study, tasks in this high regret cluster were especially likely to be quizzes and exams. Given the strong grade-based incentives for completing these tasks (of overall grade, 18 pre-lesson quizzes comprised 9%, 5 weekly review quizzes comprised 8%, 3 exams comprised 75%), this serves as practical reminder that attaching additional consequences to tasks (in this case, grades), may not make that task especially salient to the student if there are not positive motivational forces (i.e., interest, satisfaction) accompanying it.

Are there circumstances under which we might not consider it a negative thing that students do not complete a planned task? As a converse to the high-regret cluster, low cost/high satisfaction tasks (Cluster 3) represented low-cost tasks that were indeed both interesting and satisfying. Students were not especially likely to complete these tasks, but they also anticipated little regret if they failed to finish them. There are sensible reasons that a student may not complete a task that they hoped to accomplish but did not put a lot of importance in. Task reengagement can occur in numerous ways and may relate to goal hierarchies (Ntoumanis & Sedikides, 2018). For instance, a task that is rated low in priority today because it is not due may rise in priority as the deadline approaches. Failing to complete all tasks after setting an ambitious agenda is not necessarily worse than setting fewer tasks and accomplishing all of them, especially if there are few consequences for putting those tasks off.

Coding the types of tasks themselves allowed us to connect theory and practice by identifying what types of assignments were especially likely to be approached with specific profiles of motivation and emotion. As noted above, students were especially likely to characterize quizzes and exams as activities they would feel a lot of regret if not completed. However, the 26 total pre-lesson quizzes, weekly review quizzes, and exams all took place within a five-week course, were often not regarded with much interest or anticipated satisfaction, and surprisingly, were not especially likely to be completed despite constituting over 90% of students’ grade. Descriptive statistics confirmed that for any given assessment, 5–15% of students who completed the course simply did not do them. This suggests that when assessments are so plentiful, instructors may need to do more to raise students’ interest in them in order to avoid students falling behind simply because they are choosing not to do them.

We also found that discussion posts, which are often a key component of online courses, were especially likely to be approached with two quite different profiles of motivation and emotion. On the one hand, they were often approached with high emotions/low cost (Cluster 1), which were the tasks most likely to be completed. On the other hand, they were also often approached with low emotions/low cost (Cluster 6). Essentially, although students did not find discussion posts costly, some found completing them both interesting and emotionally charged whereas others felt the opposite. This reinforces the potential for divergent attitudes towards peer interaction in online courses. Recent research has suggested that students who enjoy demonstrating their abilities to their classmates realize that when in asynchronous online courses, discussion posts are one of the only ways to make their abilities visible to others (McPartlan et al., 2019). Meanwhile, some students feel that discussion posts represent a disingenuous and not very useful exercise when they attribute classmates’ participation to simply getting a good grade instead of a genuine interest in engaging in a thought-provoking discussion (McPartlan & Rutherford, 2018). Finally, the various social and cognitive costs of engaging with classmates can lead students to avoid assigned discussion posts altogether (McPartlan et al., 2020). Therefore, the results suggest that these assignments, which gave students several specific options for how to discuss the course material, did a relatively good job at lowering costs of participating, but did not always succeed in affectively engaging students. As courses rapidly move online, new research may consider how emotions and cost interact to improve online students’ engagement in the more social aspects of online learning.

### Limitations and future directions

As the study relied on longitudinal correlational data, no strong claims can be made about the causal relationships between variables. Additionally, the sample was restricted to undergraduates in an elective online course, which may limit generalizations to other age groups and achievement contexts. Although we conceptualized task values and emotions as task-specific and thus subject to change depending on the nature of the task, we operationalized effort regulation in the presurvey as a stable trait in our study. However, effort regulation may also be conceptualized as task-specific, especially for online students. Emerging research on “Zoom fatigue,” for example, suggests how the cognitive and physical demands of certain online tasks may make prolonged effort regulation especially taxing (Bailenson, 2021). We believe future studies should investigate effort regulation at the task level to investigate heterogeneity in regulating effort depending on the value of the task.

To reduce bias and the likelihood of Type I errors in multilevel logistic regression models, sample sizes with 50 level-1 units (task observations) nested within 50 to 80 level-2 units (people) have been recommended (Moineddin et al., 2007; Schoeneberger, 2016). Our sample consisted of 75 people but with a maximum of ten task observations per person. Therefore, we recommend a replication study with more task observations and individuals.

Finally, we recommend that future research on task behavior measures the specific behavior under study and whether task disengagement may be adaptive at times. It may be that ambitious students intend to do assignments and schoolwork ahead of time and strategic disengagement from a task with low cost and value may be adaptive and beneficial in order to complete other tasks that are more time sensitive and important.

## Appendix

See Table

**Table 6:** Daily academic task types by cluster

	High emotions/ high cost	Medium emotions/ medium cost	Low cost/ high satisfaction	High emotions/ low cost	High regret	Low emotions/ low cost
Quiz/exam	58	22	21	46	**31**	**5**
	51.36	28.62	23.85	45.18	**21.61**	**12.35**
	1.287	− 1.59	− 0.74	0.164	**2.535**	− **2.554**
Textbook reading	27	18	7	14	9	4
	22.17	12.35	10.29	19.5	9.33	5.331
	1.289	1.864	− 1.176	− 1.533	− 0.123	− 0.637
Textbook study guide	**20**	19	18	30	14	8
	**30.59**	17.05	14.21	26.91	12.87	7.356
	− **2.475**	0.563	1.181	0.751	0.367	0.27
Watching video lectures	59	27	32	46	**12**	13
	53.04	29.56	24.64	46.67	**22.32**	12.755
	1.143	− 0.61	1.887	− 0.134	− **2.76**	0.084
Discussion posts	**10**	11	4	**23**	4	**8**
	**16.84**	9.38	7.82	**14.81**	7.08	**4.049**
	− **2.063**	0.602	− 1.538	**2.571**	− 1.295	**2.134**
Review	9	5	3	**2**	7	**6**
	8.98	5	4.17	**7.902**	3.779	**2.16**
	0.007	− 0.003	− 0.631	− **2.481**	1.809	**2.775**

Adjusted squared residual for each cell displayed below observed and expected frequencies. Bolded cells are those in which expected values significantly differ from observed values at an alpha level of 0.05 (not corrected for multiple tests)

6.

## Footnotes

1 Interest has been conceptualized as both an emotion (Pekrun, 2006; Silvia, 2008) and as part of task value in expectancy-value research.

2 Recent research has also suggested that cost may be conceptually and empirically distinct from task-value (Flake et al., 2015; Kosovich et al., 2015).
